# Molten Salt Synthesis of High-Purity Ti_2_AlC Powders and Fabrication of Conductive Ag/Ti_2_AlC Composites

**DOI:** 10.3390/ma19071448

**Published:** 2026-04-04

**Authors:** Zheng Yue, Lisheng Cao, Jianxiang Ding, Shikun Ma, Yiming Cai, Haoyu Yang, Ruixiang Qiu, Jin Qian, Bo Li, Pengfei Feng, Wei Liu, Jinlong Wang, Chenghuan Huang

**Affiliations:** 1 School of Materials Science and Engineering, Anhui University of Technology, Ma’anshan 243002, China; yz2606280690@outlook.com (Z.Y.); 18155518127@163.com (L.C.); jxding@ahut.edu.cn (J.D.); skma0115@163.com (S.M.); cym3531672215@163.com (Y.C.); yhy0960@163.com (H.Y.); ruixiangqiu0401@163.com (R.Q.); 19055402531@163.com (J.Q.); 2 Guilin Electrical Equipment Scientific Research Institute Co., Ltd., Guilin 541004, China; fengpf_jg@163.com; 3 AMK (Anhui) Automotive E-Drive Co., Ltd., Xuancheng 242300, China; 4 Xiamen Jinbo Noble Metal Product Co., Ltd., Xiamen 361021, China; 1001934@hongfa.cn; 5 Xiamen Hongfa Electroacoustic Co., Ltd., Xiamen 361021, China; 1002604@hongfa.cn

**Keywords:** Ti_2_AlC, molten salt synthesis, reaction pathway engineering, high-purity powders, conductive composites

## Abstract

Ti_2_AlC, an important member of the MAX phase family, exhibits combined metallic and ceramic characteristics, showing potential for applications in conductive composites and high-temperature structural components. However, this phase possesses a narrow thermodynamic stability window, making high-purity synthesis challenging. Conventional solid-state synthesis requires temperatures exceeding 1300 °C, where aluminum volatilization and kinetic limitations of carbon diffusion lead to impurity phases such as TiC and Ti_3_AlC_2_. Based on the ionic transport characteristics of molten salt media, this study employed the eutectic NaCl-KCl molten salt method to synthesize Ti_2_AlC using Ti, Al, and TiC powders within the temperature range of 1000–1150 °C. Systematic investigations revealed that an optimized raw powder composition (Ti:Al:TiC = 1:1.10:0.95) at 1100 °C yielded Ti_2_AlC powders with 96.1% phase purity, high crystallinity, and typical laminated structure with stable stoichiometry (Ti/Al ≈ 2:1). Furthermore, Ag/Ti_2_AlC composites demonstrated excellent electrical conductivity (resistivity of 5.72 μΩ·cm) and favorable mechanical properties, validating the applicability of this synthetic route for conductive composite materials.

## 1. Introduction

MAX phases are a unique family of layered ternary carbides and nitrides with the general formula M_n+1_AX_n_, where M represents an early transition metal, A is an A-group element (typically from groups 13–16), and X is carbon or nitrogen [1,2,3,4]. Over the past two decades, these materials have attracted much research interest due to their combined metallic and ceramic properties, including high electrical and thermal conductivity, excellent workability, and high-temperature oxidation resistance [5,6,7,8,9]. Among the various members of this family, Ti_3_AlC_2_ has been extensively investigated for superior high-temperature oxidation resistance afforded by the formation of a protective Al_2_O_3_ scale, excellent electrical conductivity and tribological properties suitable for electrical contact applications, and good damage tolerance with machinability [10,11,12,13,14,15]. However, the isostructural Ti_2_AlC, which possesses lower density and theoretical elastic modulus, offers distinct advantages for lightweight conductive applications despite having received comparatively less attention [5].

Ti_2_AlC exhibits favorable metallic conductivity with a resistivity of approximately 3.6 × 10^−7^ Ω·m. Nevertheless, this value remains significantly higher than that of noble metals such as silver (1.6 × 10^−8^ Ω·m) [2]. Silver exhibits intrinsic high electrical and thermal conductivity, coupled with excellent chemical stability. Consequently, it has been extensively employed in high-frequency switching and miniature relay applications [16,17]. Recently, silver has also been widely investigated as a matrix material for MAX phase composites to combine its superior conductivity with the mechanical robustness of MAX phases [18].

For electrical contact applications requiring both high conductivity and wear resistance, Ag/Ti_2_AlC composites represent a promising solution. In this system, the Ag matrix provides excellent electrical pathways, while the Ti_2_AlC reinforcement enhances mechanical strength and arc erosion resistance. Previous studies on Ag/Ti_3_AlC_2_ composites have achieved resistivities as low as 5.69 × 10^−8^ Ω·m while exhibiting excellent arc erosion resistance [19]. However, Ti_2_AlC-based composites remain underexplored, primarily because the synthesis of high-purity Ti_2_AlC tailored for such applications remains challenging. Given its lower density and theoretical elastic modulus compared to Ti_3_AlC_2_, Ti_2_AlC holds significant potential for next-generation lightweight conductive composites, warranting further investigation beyond its traditional structural applications.

Ti_2_AlC maintains low electrical resistivity similar to Ti_3_AlC_2_ (~3.6 × 10^−7^ Ω·m) and good thermal conductivity. Furthermore, due to its lower aluminum content, Ti_2_AlC shows better oxidation resistance and creep resistance at elevated temperatures. These characteristics make Ti_2_AlC particularly attractive for applications requiring lightweight conductive materials, such as electrical contacts, heating elements, and conductive composite reinforcements [5,20]. Nevertheless, the synthesis of phase-pure Ti_2_AlC is difficult and has limited its practical use. Thermodynamic calculations and experimental observations indicate that Ti_2_AlC has a narrow stability window within the Ti-Al-C ternary phase diagram, typically behaving as a metastable intermediate phase rather than a thermodynamically stable end-product [21,22]. During conventional solid-state synthesis methods such as hot pressing or spark plasma sintering of elemental powders, Ti_2_AlC readily transforms into the Al-rich Ti_3_AlC_2_ phase at high temperatures or decomposes into TiC and Al-based intermetallic compounds at lower temperatures, making it difficult to precisely control the reaction at the 211-phase composition [23,24]. The formation of competing phases, particularly titanium carbide (TiC) and Al–Ti intermetallics such as TiAl_3_ and Ti_3_Al, further makes the synthesis difficult. These secondary phases not only reduce the intrinsic properties of Ti_2_AlC but also decrease the homogeneity of the reinforcement phase in composite materials, directly reducing the reliability of the material under demanding service conditions [21]. The kinetic barriers associated with solid-state diffusion require high processing temperatures, typically exceeding 1300 °C, which often lead to aluminum loss and subsequent stoichiometric deviations that push the system toward TiC or Ti_3_AlC_2_ formation [22,23,25]. Consequently, conventional preparation methods often produce multiphase products containing substantial TiC impurities, limiting the ability to isolate and study the intrinsic properties of phase-pure Ti_2_AlC.

To circumvent these thermodynamic and kinetic constraints, molten-salt-assisted synthesis represents an effective alternative strategy for the low-temperature preparation of MAX phases and their derivative MXenes [26,27]. Unlike conventional solid-state sintering, the molten salt method utilizes the molten salt medium to enable nucleation and growth of the target phase at lower temperatures, effectively suppressing the formation of high-temperature secondary phases [28]. Among various salt systems, the eutectic NaCl-KCl mixture is commonly selected as the primary molten salt medium due to its advantageous combination of low cost, low melting point (~658 °C), and high chemical stability. Compared with pure NaCl (801 °C) and KCl (770 °C), this eutectic composition provides a low-viscosity liquid reaction environment at 900–1200 °C that significantly facilitates solute diffusion and energy transfer. Moreover, NaCl-KCl demonstrates excellent chemical inertness, neither reacting with reactants nor contaminating the target product, and can be easily removed by simple aqueous washing. Liu et al. employed a ball-milling-free molten salt method to synthesize high-purity Ti_3_AlC_2_ nanoparticles (~100 nm) at 900–1000 °C [29]. Moreover, Galvin et al. employed a eutectic NaCl-KCl salt to prepare Ti_2_AlC powders at 900–1000 °C; although the synthesis temperature was significantly reduced compared to traditional methods, the product phase purity reached only 88%, with residual TiC and Ti_3_AlC_2_ impurities proving difficult to eliminate [27].

In this study, we employ the eutectic NaCl-KCl molten salt method to synthesize Ti_2_AlC powders using Ti, Al, and TiC powders as raw materials at low-temperature range of 1000–1150 °C. To address the insufficient phase purity (88%) and multiphase coexistence issues, this work employs TiC rather than elemental carbon as the carbon source, which not only minimizes Ti-C side reactions but also enables reduced synthesis temperatures, thereby effectively inhibiting the formation of Ti-rich intermetallic compounds. Systematic investigation was conducted into the effects of temperature and TiC content on phase composition and microstructure, while the in situ reaction mechanism between TiC and Al in the molten salt medium were elucidated. Ultimately, Ti_2_AlC powders with phase purity exceeding 96% were successfully synthesized under controlled conditions and employed to fabricate Ag–matrix composites, demonstrating their potential as conductive reinforcements for electrical contact applications.

## 2. Materials and Methods

### 2.1. Molten Salt Synthesize Ti_2_AlC with Different Al Content

Powders of Ti (Aladdin, Shanghai, China; ≥99.99%), Al (Aladdin, Shanghai, China; ≥99.99%), and TiC (Aladdin, Shanghai, China; ≥99%) were weighed according to the nonstoichiometric mole ratio of 1Ti/xAl/0.95TiC (x varying from 1.00 to 1.15) to systematically investigate the effect of Al content on the phase formation and purity of Ti_2_AlC. The detailed weighing parameters for each composition are listed in Table 1. The raw powder mixture was homogenized in a three-dimensional mixer (Turbula T2F, WAB, Muttenz, Switzerland) for 24 h, and subsequently mixed with 20 g of pre-dried NaCl-KCl eutectic salt (molar ratio 1:1; NaCl: AR, ≥99.5%; KCl: GR, ≥99.8%; Aladdin, China) in a mass ratio of 1:10 (powder to salt) within an alumina crucible. The crucible was placed in a graphite boat and heated in a tube furnace to 1100 °C at a heating rate of 7 °C/min, held for 1 h under flowing argon (>99.999%), and then naturally cooled to room temperature. The resulting product was washed repeatedly with boiling deionized water to remove residual salts, and finally dried at 55 °C for 12 h. Typically, approximately 1.8 g of Ti_2_AlC powder was obtained from the raw materials, corresponding to a mass yield of about 90%.

### 2.2. Molten Salt Synthesize Ti_2_AlC with Different Holding Temperature

The NaCl-KCl mixture and raw material powder were mixture prepared as described in Section 2.1. To investigate the influence of holding temperature on phase formation and purity, the samples were separately heated to 1000 °C, 1050 °C, 1100 °C and 1200 °C at 7 °C /min, held for 1 h under flowing argon (>99.999%), and then furnace-cooled. Subsequent washing with boiling deionized water and drying (55 °C for 12 h) followed the procedures described in Section 2.1.

### 2.3. Preparation of Ag/Ti_2_AlC Composite

The as-prepared Ti_2_AlC powder was mixed with silver powder at a weight ratio of 1:9 (Ti_2_AlC = 2 g, Ag = 18 g). The mixed powder was then subjected to wet ball milling with anhydrous ethanol and zirconia balls at a mass ratio of 1:1:1.5. The ball milling was conducted at a rotation speed of 450 r/min for 30 min. The resulting slurry was dried at 55 °C for 24 h and subsequently sieved to obtain the Ag/Ti_2_AlC precursor powder. Finally, 15 g of the mixed powder was compacted into bulk material by hot press at 120 °C under the pressure of 550 MPa for 5 min. Finally, the bulk specimens were heat-treated at 800 °C for 2 h and subsequently furnace-cooled.

### 2.4. Characterization

Macro-morphologies of flake samples before and after testing were observed by the digital camera (6D Mark II, Canon, Tokyo, Japan). The phase constitutions were examined by X-ray diffraction (XRD, AXS D8, Bruker, Karlsruhe, Germany). Micro-morphologies were observed by a field emission scanning electron microscope (FEI-SEM, Nova NanoSEM 450, FEI company, Hillsboro, OR, USA), and their chemical elements distribution were analyzed by an energy dispersive spectrometer (EDS, AZtec X-MaxN80, Oxford Instruments, Abingdon, UK). The density of Ag/10 wt.% Ti_2_AlC flake samples were tested with the densimeter (AR-150PM, DahoMeter, Dongguan, China). The resistance of flake samples (dimensions: 15 mm × 1.5 mm × 1.0 mm) was obtained by a high-precision milliohm instrument (METRAHIT 27 I, Gossen Metrawatt, Nuremberg, Germany, 0.001 mΩ accuracy), then the resistivity was calculated according to the formula ρ = *R·S/L*, where *R* is the measured resistance, *S* is the cross-sectional area perpendicular to the current direction (width × thickness), and *L* is the actual probe spacing. Details of the test setup are shown in Figure S1 of the Supplementary Information. All flake samples were cut into dumbbell-shaped specimens (gauge dimensions detailed in Supplementary Figure S2) for tensile strength testing according to GB/T 228.3-2019 [30] (equivalent to ASTM E8 [31] for thin metallic sheets), using a universal test machine (AGS-X5kN, SHIMADZU, Tokyo, Japan) at a crosshead speed of 1 mm·min^−1^.

## 3. Results and Discussion

**Figure 1:**
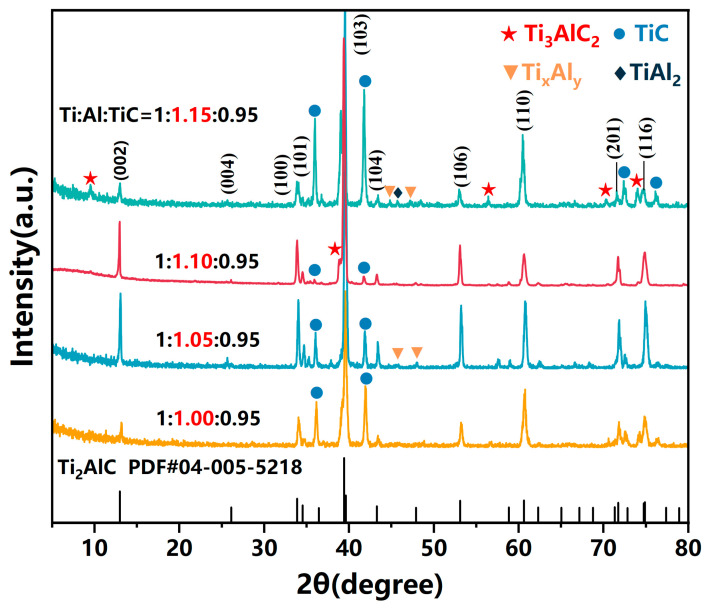
XRD result of Ti_2_AlC with different raw powder molar ratios.

Figure 1 presents the X-ray diffraction (XRD) patterns of products synthesized with varying Ti:Al:TiC molar ratios (Al content increasing from 1.00 to 1.15). Comparison with the standard reference pattern of Ti_2_AlC confirms that the principal diffraction peaks in all samples correspond to the target phase. At an Al content of 1.00, the characteristic peaks of Ti_2_AlC exhibit relatively low intensities, accompanied by significant signals from impurity phases (TiC and other intermediates), indicating incomplete reaction due to insufficient formation of TiAl intermetallic precursors [24]. As the Al content increases to 1.05, diffraction peaks corresponding to TiC remain identifiable. At an Al content of 1.10, the Ti_2_AlC peak intensities increase significantly, demonstrating high crystallinity with minimal secondary phases, which suggests that this stoichiometry satisfies the requirements for Ti_2_AlC formation while effectively compensating for Al volatilization losses during sintering [27]. Further increasing the Al content to 1.15, the diffraction peak intensities of Ti_3_AlC_2_ and TiC increase markedly, and peaks corresponding to Ti_x_Al_y_ intermetallic compounds emerge in the 2θ range of approximately 43–45°. This observation is consistent with previously reported reaction mechanisms, wherein an Al-rich environment promotes the formation of Ti_3_AlC_2_ and Al-rich intermetallic phases [25].

**Figure 2:**
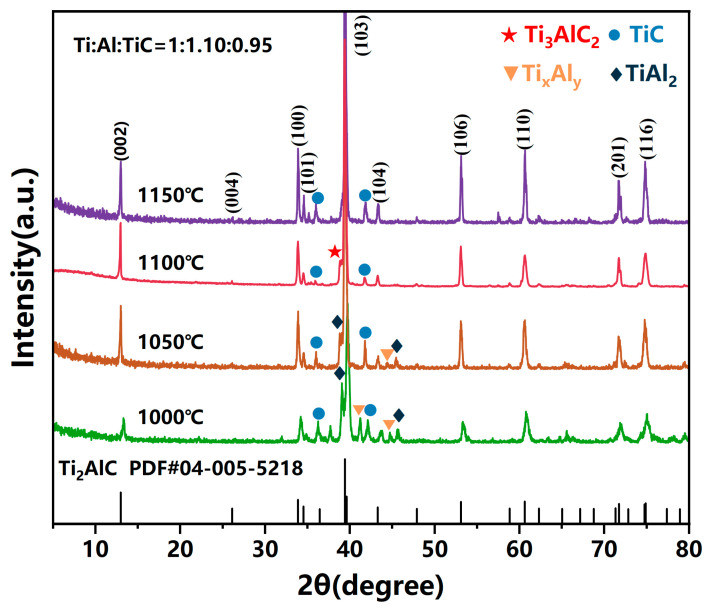
XRD result of Ti_2_AlC with different holding temperature.

Figure 2 presents the XRD patterns of samples synthesized at various temperatures with a fixed precursor ratio of Ti:Al:TiC = 1:1.10:0.95. At 1000 °C and 1050 °C, distinct diffraction peaks corresponding to TiAl_2_ and Ti_x_Al_y_ intermetallic compounds are observed alongside the Ti_2_AlC phase, indicating incomplete solid-state reactions where Al and Ti preferentially formed thermodynamically favorable intermediate phases rather than the layered carbide. At 1100 °C, these intermetallic peaks vanish, yielding phase-pure Ti_2_AlC with high crystallinity. However, increasing the temperature to 1150 °C results in the emergence of TiC peaks, attributable to Al volatilization at excessive temperatures, which disrupts the stoichiometric stability and induces decomposition to TiC. Moreover, the content of Ti_2_AlC was calculated according to the following formula [32]:

(1) $$W=\frac{I_{b}}{4.545I_{a}+I_{b}+0.382I_{c}}$$

where *I_a_*, *I_b_*, and *I_c_* represent the integrated intensities of the Ti_3_AlC_2_ (002), Ti_2_AlC (002), and TiC (111) diffraction peaks, respectively. Ultimately, the calculated purity of the sample synthesized at 1100 °C was determined to be 96.1%. The phase purity calculated by Equation (1) is further validated by Rietveld refinement analysis (Figure S3, Supplementary Information). The refinement confirms the as-synthesized powder contains 96.1 wt.% Ti_2_AlC with only 3.9 wt.% TiC impurity, exhibiting a low weighted profile R-factor (R_wp_ = 14.92%). The consistency between the two methods confirms the high reliability of the phase purity assessment. The trace TiC content (<4%) is attributed to the intrinsic narrow thermodynamic stability window of Ti_2_AlC and incomplete reaction of the TiC precursor, which is consistent with previously reported molten salt synthesis studies.

**Figure 3:**
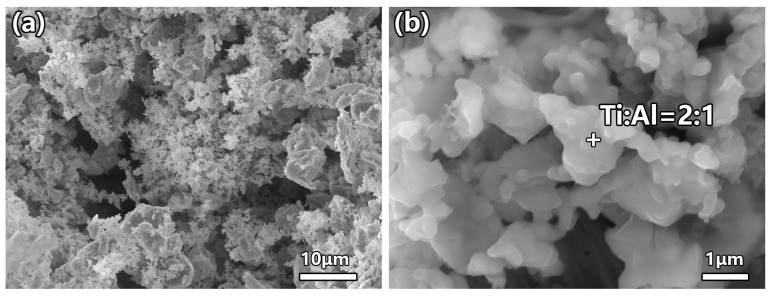
SEM images of as-synthesized Ti_2_AlC: (**a**) low-magnification overview; (**b**) high-magnification detail with EDS analysis (the cross symbol “+” indicates the location of EDS analysis).

Figure 3 presents the SEM morphologies of Ti_2_AlC powder synthesized at 1100 °C using a precursor ratio of Ti:Al:TiC = 1:1.10:0.95. Figure 3a reveals that the powder primarily consists of irregular flaky particles with relatively uniform distribution, exhibiting particle sizes ranging from several micrometers to over ten micrometers. Some fine particles adhere to the surfaces of larger ones, displaying typical agglomeration characteristics associated with molten salt-assisted synthesis. The high-magnification SEM image (Figure 3b) clearly demonstrates the lamellar structure on particle surfaces, consistent with the crystallographic features of the Ti_2_AlC MAX phase. Energy-dispersive spectroscopy (EDS) analysis (Figure 3b) indicates an atomic ratio of Ti to Al of approximately 2:1 in this region, matching the theoretical stoichiometry of Ti_2_AlC. This suggests that at 1100 °C, aluminum volatilization is effectively suppressed, and sufficient atomic diffusion supports complete solid-state reactions, resulting in fully developed grains with high crystallinity. These observations corroborate the sharp diffraction peaks and high crystallinity observed in the XRD patterns.

**Figure 4:**
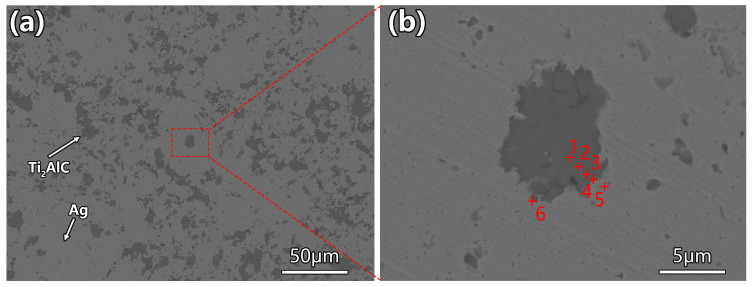
SEM images of Ag-Ti_2_AlC composite: (**a**) overview; (**b**) high-magnification detail with EDS points (the cross symbol “+” indicates the location of EDS analysis).

Ti_2_AlC particles are uniformly dispersed throughout the silver matrix without obvious agglomeration as shown in Figure 4a, indicating that the wet ball milling and hot pressing processes achieved effective distribution of the filler within the matrix. Figure 4b shows a higher-magnification view of a selected individual Ti_2_AlC particle and its surrounding region subjected to micro-area compositional analysis, with the results summarized in Table 2.

The EDS analysis reveal that Points 1–3 are situated within the particle interior, exhibiting Ti/Al atomic ratios of 2.67, 2.45, and 2.68, respectively, which are close to the theoretical stoichiometry of Ti_2_AlC. This indicates that the Ti_2_AlC particles retained their chemical stability during composite preparation without significant decomposition or reaction with the silver matrix. However, minor amounts of Ag (<5 at.%) were detected at these points, which is attributed to localized diffusion from the silver matrix during hot pressing and edge effects during EDS acquisition. Point 4 is located at the particle–matrix transition zone, where the Ti/Al ratio decreases to 1.85 and the Ag content increases significantly to 49.91 at.%, suggesting the presence of mutual elemental diffusion across the Ag/Ti_2_AlC interface. Points 5 and 6 are situated within the matrix region, showing negligible Ti and Al contents with Ag approaching 100%, confirming that the matrix consists of pure silver.

**Figure 5:**
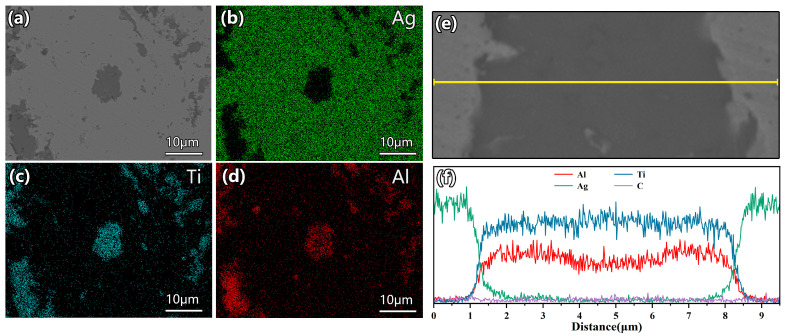
EDS analysis of the Ti_2_AlC-Ag composite (**a**) SEM image, (**b**–**d**) elemental mapping, (**e**) SEM of scan across the interface, and (**f**) EDS line scan result.

Figure 5 presents the SEM image of the Ag/Ti_2_AlC composite and the corresponding EDS elemental mapping and line scan analysis results. To evaluate the overall distribution of Ti_2_AlC particles in the Ag matrix, low-magnification SEM imaging and EDS elemental mapping were first conducted (see Figure S4 in the Supplementary Materials), confirming uniform dispersion without obvious agglomeration prior to the detailed interfacial characterization. The elemental distribution maps (Figure 5b–d) are highly consistent with the previous point analysis results. The Ag signal (Figure 5b) exhibits a continuous network-like distribution with uniform and high intensity throughout the region, indicating the formation of a well-developed conductive skeleton of the silver matrix. The signals of Ti (Figure 5c) and Al (Figure 5d) are strictly confined to the Ti_2_AlC particle regions, with their spatial distributions showing high overlap, confirming that these particles constitute Ti_2_AlC phase with stable stoichiometry. To further quantitatively characterize the interfacial features between the two phases, an EDS line scan was performed along the yellow line indicated in Figure 5e, with the results shown in Figure 5f. It can be observed that the signal intensities of Ag, Ti, and Al exhibit steep changes at the interface, with the interfacial transition zone between Ag and Ti_2_AlC being less than 0.5 μm. Such steep concentration gradients indicate that no significant elemental interdiffusion or reaction layer formation occurred between Ag and Ti_2_AlC at the heat treatment temperature of 800 °C, suggesting that the interface is dominated by physical bonding rather than metallurgical bonding.

As summarized in Table 3, the electrical resistivity of the as-prepared Ag/10 wt.% Ti_2_AlC composite (5.72 μΩ·cm) is comparable to that of the Ag/Ti_3_AlC_2_ system (5.69 μΩ·cm) and significantly lower than those of Ag/Ti_2_SnC (8.65 μΩ·cm) and Ag-TiB_2_-Ni (6.91 μΩ·cm) composites reported in the literature [19,33,34]. Although the tensile strength (149 MPa) is lower than that reported in some studies (239.65 MPa) due to the lower relative density (~95%) and physical bonding nature at the Ag/Ti_2_AlC interface (Figure 5f), it remains comparable to other Ag/MAX phase materials (Table 3), with such variations primarily arising from differences in densification temperature and interfacial bonding mechanisms—whereas higher-temperature processing typically yields stronger metallurgical bonding and higher density. These findings indicate that the Ag/Ti_2_AlC composite fabricated in this work possesses considerable potential for electrical contact applications, although further optimization of both mechanical and electrical properties is still required. Compared to Ti_3_AlC_2_, Ti_2_AlC offers lower density and theoretical elastic modulus, presenting distinct advantages for lightweight applications. Future efforts may focus on interfacial engineering and compositional regulation of the Ti_2_AlC reinforcement to achieve synergistic enhancement of the composite properties.

## 4. Conclusions

In this study, high-purity Ti_2_AlC powder (96.1% purity) was successfully synthesized via a molten salt-assisted synthesis route at 1100 °C using an optimized non-stoichiometric precursor ratio of Ti:Al:TiC = 1:1.10:0.95. This approach effectively suppressed the formation of impurity phases such as Al-Ti intermetallic compounds (TiAl_2_ and Ti_x_Al_y_), yielding Ti_2_AlC particles with well-developed lamellar structures, stable stoichiometry (Ti/Al ≈ 2:1), and high crystallinity. Building upon this, the as-synthesized high-quality Ti_2_AlC was incorporated as a reinforcement phase into a silver matrix; the resulting Ag/ Ti_2_AlC composite exhibited excellent electrical conductivity (resistivity of 5.72 μΩ·cm), validating the application potential of MAX phase powders derived from this synthetic route in conductive composite materials. This work provides a reliable experimental basis for the controlled preparation of high-quality Ti_2_AlC and its application in metal matrix composites.

## Figures and Tables

**Table 1:** Weighing parameters of raw materials for preparing Ti_2_AlC with different Al contents.

Ti/g	Al/g	TiC/g	Molar Ratio	Total/g
0.727	0.41	0.864	1:1:0.95	2
0.719	0.426	0.855	1:1.05:0.95	2
0.712	0.442	0.846	1:1.10:0.95	2
0.705	0.457	0.838	1:1.15:0.95	2

**Table 2:** EDS point results of the Ag/Ti_2_AlC composite (at.%).

Position	Ti	Al	C	Ag	Ti/Al
Point 1	60.76	22.72	11.87	0.93	2.67
Point 2	57.99	23.65	15.97	2.38	2.45
Point 3	57.62	21.47	16.34	4.57	2.68
Point 4	17.53	9.50	13.59	49.91	1.85
Point 5	0	0	0	100	/
Point 6	0	0	0	100	/

**Table 3:** Comparison of properties of typical Ag/conductive ceramic electrical contact composites.

Material	Resistivity (μΩ·cm)	Tensile Strength (MPa)	Reference
Ag/10 wt.% Ti_2_AlC	5.72	149.00	This work
Ag/10 wt.% Ti_3_AlC_2_	5.69	172.10	[19]
Ag/10 wt.% Ti_2_SnC	8.65	134.56	[33]
Ag/10 wt.% Ti_2_AlC	6.71	239.65	
Ag/4 wt.% TiB_2_–4 wt.% Ni	6.91	404.69	[34]

## Data Availability

The original contributions presented in this study are included in the article/Supplementary Material. Further inquiries can be directed to the corresponding authors.

## Supplementary Materials

The following supporting information can be downloaded at: https://www.mdpi.com/article/10.3390/ma19071448/s1, Figure S1: Dimensions of resistivity measurement specimen and photograph of set up; Figure S2: Dimensions of dumbbell-shaped tensile specimen; Figure S3: Rietveld refinement of XRD pattern for as-synthesized Ti_2_AlC powder; Figure S4: Low-magnification SEM image and EDS elemental maps of Ag/Ti_2_AlC composite.
