# Supplementary material for: Molten Salt Synthesis of High-Purity Ti2AlC Powders and Fabrication of Conductive Ag/Ti2AlC Composites
Source: Materials (Basel). 2026 Apr 4;19(7):1448. doi: 10.3390/ma19071448 (PMC13074225; doi:10.3390/ma19071448)
Supplement: Supplementary file 1 [file materials-19-01448-s001.zip › materials-4217641-supplementary.pdf]

Supplementary Information

Molten salt synthesis of high-purity Ti_2AlC powders and fabrication of conductive Ag/Ti_2AlC composites

Zheng Yue [1,†], Lisheng Cao[1,†], Jianxiang Ding [1], Shikun Ma[1], Yiming Cai[1], Haoyu Yang[1], Ruixiang Qiu[1], Jin Qian[1], Bo Li [2,*], Pengfei Feng[2], Wei Liu [3,*], Jinlong Wang [4], Chenghuan Huang [5]

Characterization:

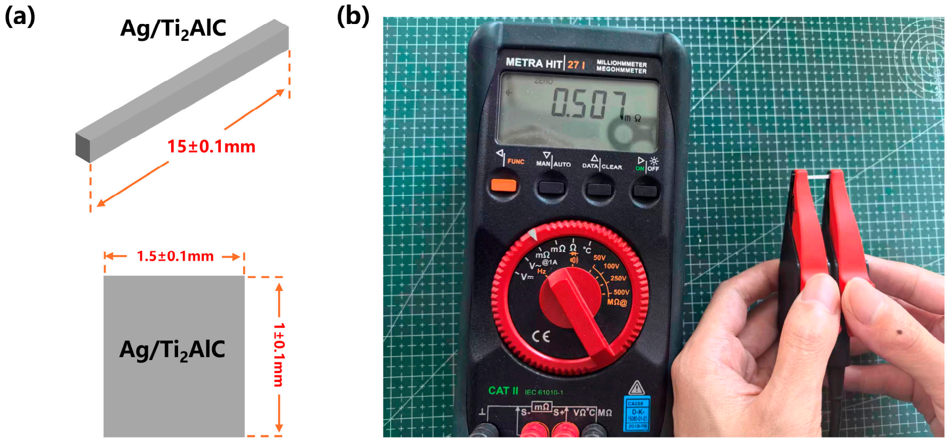

Figure S1. (a) Schematic illustration showing the dimensions of the Ag/Ti_2AlC composite specimen used for electrical resistivity measurements; (b) Photograph of the four-point probe measurement setup.

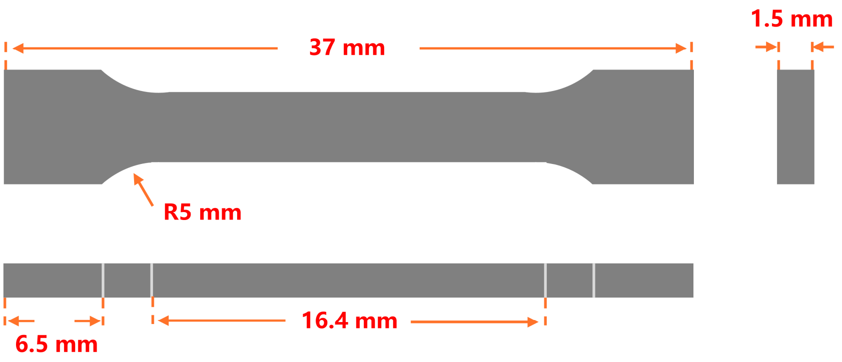

Figure S2. Schematic illustration showing the dimensions of the dumbbell-shaped tensile specimen.

Phase Analysis:

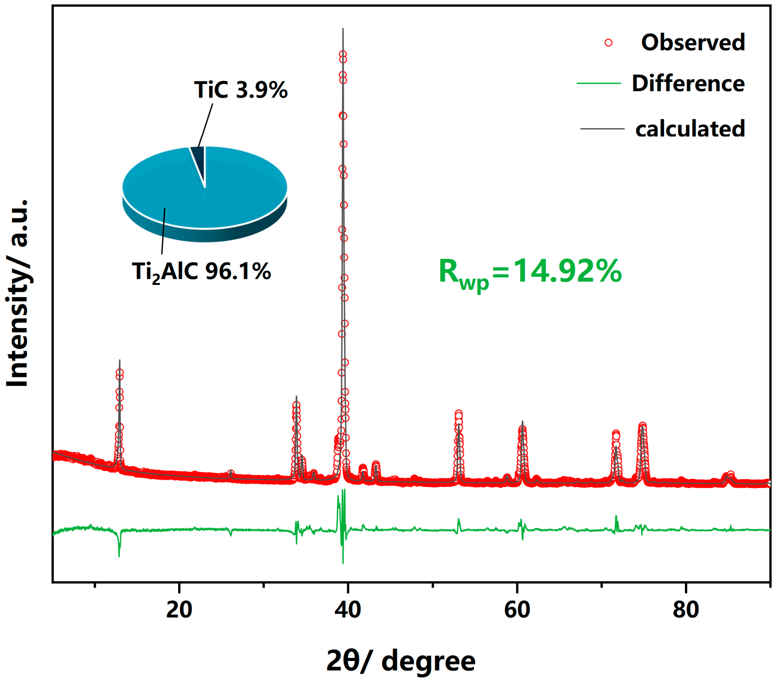

Figure S3. Rietveld refinement of the XRD pattern for the as-synthesized Ti_2AlC powder

Rietveld refinement analysis confirms that the as-synthesized Ti_2AlC powder exhibits high phase purity, with the target Ti_2AlC phase accounting for 96.1% and only a minor impurity of TiC (3.9%) detected (Figure S1). The relatively low weighted profile R-factor (R_{wp} = 14.92%) indicates good agreement between the observed and calculated patterns, validating the reliability of the refinement results. The presence of trace TiC (<4%) is attributed to the intrinsic narrow thermodynamic stability window of Ti_2AlC and incomplete reaction of the TiC precursor, which is consistent with previously reported molten salt synthesis studies. Overall, the quantitative phase analysis demonstrates the effectiveness of the optimized synthesis parameters (1100°C, Ti:Al:TiC = 1:1.10:0.95) in achieving high-purity Ti_2AlC suitable for composite reinforcement applications.

Microstructural Characterization:

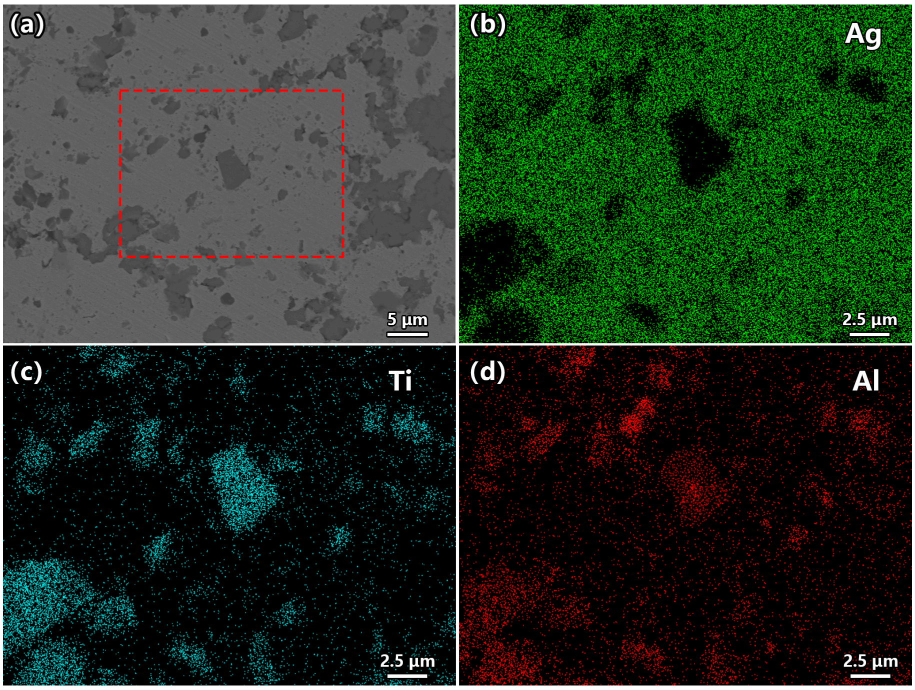

Figure S4 (a) Low-magnification SEM image and (b-d) EDS elemental maps of the Ag/Ti_2AlC.

Low-magnification SEM and EDS mapping reveal that Ti_2AlC particles are uniformly dispersed throughout the Ag matrix without agglomeration (Figure S2a), with EDS confirming the continuous Ag network and discrete localization of Ti and Al within the reinforcement phase (Figure S2b-d).
